# Discovery of a giant pulmonary hydatid cyst on thoracic deformation in a child

**DOI:** 10.11604/pamj.2022.42.39.35101

**Published:** 2022-05-16

**Authors:** Hind Cherrabi, Mohamed Amine Oukhouya

**Affiliations:** 1 Faculty of Medicine and Pharmacy, Ibn Zohr University, Agadir, Morocco

**Keywords:** Hydatid cyst, lung, giant, child, thoracic deformation

## Image in medicine

A 9-year-old girl without notable history presented with a right thoracic deformity in the context of apyrexia. On admission, she was hemodynamically stable, not dyspneic with no notion of hemoptysis or hydatid vomiting. The right hemithorax was bulging on inspection, associated with a right fluid effusion syndrome. The radiograph showed: the right pleurisy of great abundance, pushing back the trachea. The thoracic computed tomography (CT) scan: a giant hydatid cyst measuring 20 cm x 13 cm of great axis of the middle and upper lobar, associated with reactive pleurisy. The resection of the protruding dome and the extraction of the proligeral membranes were carried out. Blinding of the fistulas was continued by padding. After the stay in intensive care, respiratory physiotherapy was started. The postoperative course was simple, the control X-ray showed the re-expansion of the lung at the wall.

**Figure 1:**
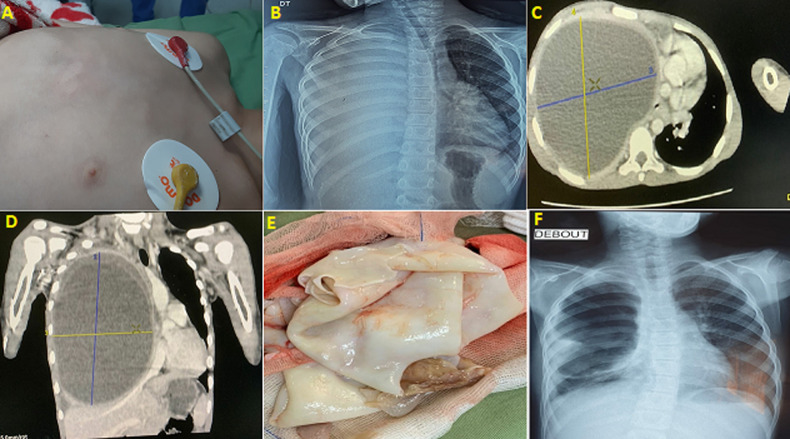
A) preoperative image showing the bulging aspect of the right hemithorax; B) standard radiograph showing the appearance of right pleurisy; C) computed tomography scan in axial sections showing the right huge; D) computed tomography scan in frontal sections showing the right huge; E) intra-operative image of the proligeral membranes; F) standard control radiograph showing the re-expansion of the right lung

